# Development of a relevant strategy using de novo transcriptome assembly method for transcriptome comparisons between Muscovy and common duck species and their reciprocal inter-specific mule and hinny hybrids fed ad libitum and overfed

**DOI:** 10.1186/s12864-020-07099-4

**Published:** 2020-10-02

**Authors:** Xi Liu, Frédéric Hérault, Christian Diot, Erwan Corre

**Affiliations:** 1 ABiMS Bioinformatics Facility, CNRS, Sorbonne Université, FR2424, Station Biologique, 29680 Roscoff, France; 2 UMR PEGASE, INRAE, Institut Agro, 16 Le Clos, 35590 Saint-Gilles, France

**Keywords:** RNA sequencing, Interspecific hybrids, De novo transcriptome assembly, Gene expression, Liver steatosis

## Abstract

**Background:**

Common Pekin and Muscovy ducks and their intergeneric hinny and mule hybrids have different abilities for fatty liver production. RNA-Seq analyses from the liver of these different genetic types fed ad libitum or overfed would help to identify genes with different response to overfeeding between them. However RNA-seq analyses from different species and comparison is challenging. The goal of this study was develop a relevant strategy for transcriptome analysis and comparison between different species.

**Results:**

Transcriptomes were first assembled with a reference-based approach. Important mapping biases were observed when heterologous mapping were conducted on common duck reference genome, suggesting that this reference-based strategy was not suited to compare the four different genetic types. De novo transcriptome assemblies were then performed using Trinity and Oases. Assemblies of transcriptomes were not relevant when more than a single genetic type was considered. Finally, single genetic type transcriptomes were assembled with DRAP in a mega-transcriptome. No bias was observed when reads from the different genetic types were mapped on this mega-transcriptome and differences in gene expression between the four genetic types could be identified.

**Conclusions:**

Analyses using both reference-based and de novo transcriptome assemblies point out a good performance of the de novo approach for the analysis of gene expression in different species. It also allowed the identification of differences in responses to overfeeding between Pekin and Muscovy ducks and hinny and mule hybrids.

## Background

In waterfowls, liver steatosis can occur spontaneously as a result of energy storage before migration. This natural ability has been exploited for thousand years to produce fatty liver or “foie gras” by overfeeding ducks or geese in a short period of time. However, fatty liver production varies according to species and breeds [1–5]. In France, the main producer worldwide, most of the production (90%) involves Mule ducks, i.e. intergeneric hybrids between a female common Pekin duck (*Anas plathyrynchos*) and a male Muscovy duck (*Cairina moschata*), and in a far lesser extent Muscovy ducks or geese from the Landes grey breed (*Anser anser*). Conversely, Pekin ducks are not involved due to their lower ability to produce fatty liver. Some studies have been conducted to better characterize hepatic steatosis development in waterfowls and analyze differences between genotypes. However, they mainly focused on biochemical levels [1–5]. Some studies also focused on gene expression levels but were conducted on few candidate genes only [6–9]. This was mainly due to the lack of duck specific microarrays for transcriptome analyses at the genome level. Thus, genome-wide analyses of gene expression are still needed to better characterize hepatic steatosis in ducks and their hybrids.

The recent development of high throughput RNA sequencing now allows characterization of expressed transcripts and quantification in samples of any species without the need of preexisting tools such as microarrays [10, 11]. Different strategies were applied for transcriptome assembly and analyses of RNA sequences referred as reference-based or de novo assemblies [12–15]. Reference-based strategy is generally conducted when a reference genome for the target transcriptome is available. In such a situation, RNA sequences are aligned on the reference genome using mapping tools like Burrows-Wheeler transform (BWA) [16] or TopHat-Cufflinks [17]. This approach has also been applied in heterologous situations where reference genome and RNA sequences were from different species, for examples mapping horse, donkey and their hybrids transcriptomes on horse reference genome [18], sparrows transcriptome on zebra finch reference genome [19] or red deer transcriptome on cow reference genome [20]. In a previous study we have also conducted such a strategy on common and Muscovy ducks and their interspecific hybrids using the common duck genome as reference [21]. This strategy was relevant to analyze differences in gene expression within a genetic type as reads from the same type would have the same mapping rate. However, a bias was expected in read mapping and counting according to different homologous, “half-homologous” and heterologous situations making this strategy not relevant to analyze differences in gene expression between genetic types. In such heterologous situations, i.e. in the absence of reference genome from the same species or when different species and hybrids are compared, de novo approaches are more generally conducted using de novo transcriptome assembling tools such as Trinity [22] or Oases [23]. Some examples of transcriptome analyses in interspecific hybrids are the studies conducted in *cyprinidae* [24] or in brassica [25]. As different tools are available for reconstructing transcripts de novo, the choice of an optimal method is challenging. Surprisingly, different de novo assembly tools and methods were compared using common duck RNA sequences [26] while common duck reference genome is available [27]*.* Although different strategies can be applied to analyze transcriptomes from different species, no one appears as the best one. Interestingly, a de novo RNA-Seq Assembly Pipeline (DRAP) has been developed improving de novo transcriptome assemblies performed by Trinity and Oases in terms of compaction (number of contigs needed to represent the transcriptome) and quality (chimera and nucleotide error rates) [28].

In the present study, reference-based approach using *Anas platyrhynchos* genome as reference and de novo assembly approach were used and compared to analyze hepatic gene expression of overfed and ad libitum fed Muscovy and Pekin ducks and their reciprocal inter-specific Mule and Hinny hybrids and to identify differentially expressed genes between feeding and duck genetic types.

## Results

### Transcriptome assembly using reference genome

Transcriptome assemblies of RNA sequences from Pekin (*Anas platyrhynchos)*, Muscovy (*Cairina moschata)*, Hinny and Mule ducks were performed as in a previous study [21] by a reference-based approach using the common duck BGI_duck_1.0 assembly as reference genome. Heterologous mapping rate of Muscovy and to a lesser extend of Hinny and Mule transcriptomes (40, 58, 59%, respectively) were lower than homologous mapping rate of Pekin transcriptome (71%) on this common duck reference genome, clearly indicating an important bias in heterologous mapping when compared to homologous mapping. Direct mapping of reads on reference genome produced similar results (data not shown). This mapping bias prompted us to test the relevance of a de novo approach.

### De novo transcriptome assemblies

After adapters removing, quality trimming and filtering of reads, 545,691,171, 504,591,419, 534,967,150 and 627,575,995 pairs were conserved for Pekin, Muscovy, Hinny and Mule ducks, respectively. Subsequently, 37,073,089, 36,822,075, 43,650,600 and 46,866,094 pairs were selected during normalization. They were assembled de novo using Trinity in four independent “single genetic type” transcriptomes, in a “mixed parental” transcriptome (with Pekin and Muscovy reads) and in a “mixed hybrids” transcriptome (with Hinny and Mule reads). A mixed hybrids transcriptome was also assembled using Oases for comparison. As shown in Table 1, single parental species Pekin or Muscovy transcriptome assemblies with Trinity were of higher quality when compared to single hybrid Hinny or Mule transcriptome assemblies with greater average lengths, higher N50 values and lower numbers of transcripts. The quality of the mixed parental (Pekin+Muscovy) transcriptome assembly was also lower, similar to those of single hybrids transcriptome assemblies. The quality of the mixed hybrids (Hinny+Mule) transcriptome assembly was even lower. When mixed hybrids transcriptome assembly was conducted with Oases, very long transcripts with a great N50 value were produced suspected to be chimeras.

**Table 1:** De novo assemblies of transcriptomes using Trinity and Oases

Transcriptome	Pekin	Muscovy	Hinny	Mule	Pekin + Muscovy	Hinny + Mule	Hinny + Mule
Assembler	Trinity	Trinity	Trinity	Trinity	Trinity	Trinity	Oases
Transcripts	491,089	481,855	631,041	661,646	808,211	1,388,585	1,328,985
Average length	1033	1134	825	838	833	626	2532
N50	2330	2694	1441	1464	1406	858	5014

**Fig. 1:**
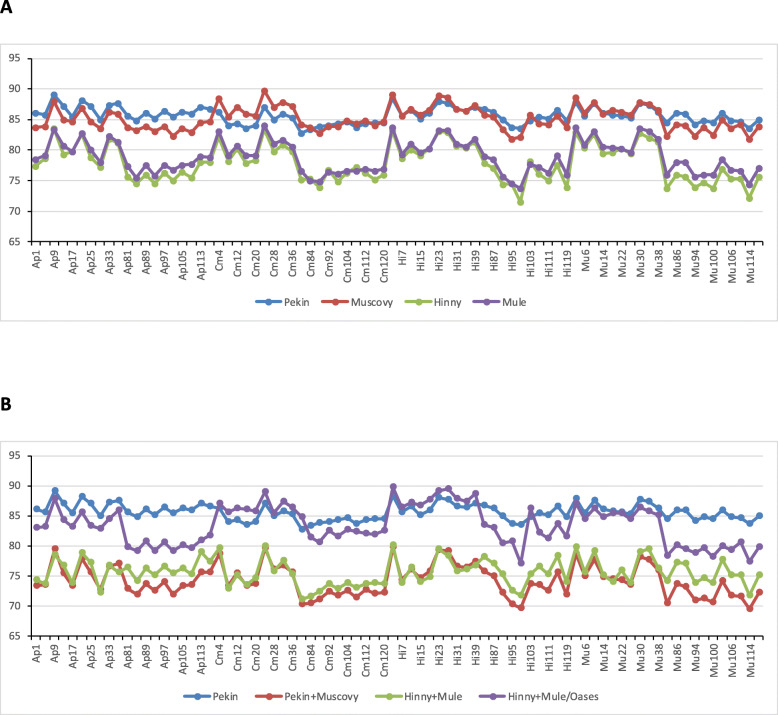
Pseudo-alignments rates of sample reads on transcriptome assemblies and quantification using Kallisto. Mapping rates of sample reads on **a** single genetic type transcriptome assemblies using Trinity or **b** on two genetic type transcriptome assemblies using Trinity (Pekin+Muscovy and Hinny+Mule) or Oases (Hinny+Mule/Oases). Pekin mapping rates in (**b**) is the same as in (**a**)

Pseudo-alignments of sample reads on these de novo transcriptome assemblies and quantification were performed using Kallisto (Fig. 1). Mapping rates of sample reads on single parental species Pekin and Muscovy Trinity assemblies were better than those on hybrids Mule and Hinny assemblies (85.63 ± 1,10%, 85.15 ± 2.27%, 77.94 ± 2.56% and 78.82 ± 2,27%, respectively) (Fig. 1a). Mapping rates on single species transcriptomes were globally better than those on mixed Pekin+Muscovy and Hinny+Mule assemblies (74.24 ± 2.14 and 75.59 ± 1.79), except for Oases assembly that displayed a similar rate but a greater variability across samples (83.43 ± 2.78%) (Fig. 1b).

**Fig. 2:**
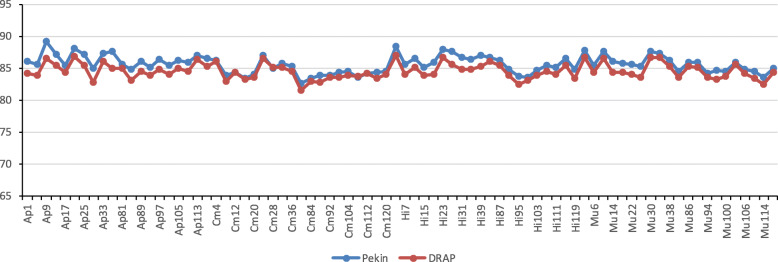
Pseudo-alignment rates of sample reads on DRAP meta-assembly. Mapping rates of sample reads on DRAP meta-transcriptome assembly. Pekin assembly using Trinity is again indicated to ease the comparison

Unique meta-transcriptomes were finally assembled with DRAP using single genetic type Pekin, Muscovy, Mule and Hinny assemblies and after filtering at 4 different FPKM thresholds. This filtering on FPKM values resulted in a reduction of transcript number by removing lowly expressed transcripts. For example, 175,572 sequences were conserved after filtering of FPKM > 1 and 64,862 with FPKM > 3, representing 286,879 845 and 127,395,003 residues, respectively. We chose to keep the transcriptome assembly produced by DRAP after FPKM > 1 filtering for further analyses. This assembly was of high quality, with a N50 value of 2807 bp. Pseudo-alignments of reads on this meta-transcriptome and quantification were performed using Kallisto. As shown on Fig. 2, pseudo-alignment rates of sample reads on DRAP meta-assembly were very similar to pseudo-alignment rates on Pekin Trinity assembly, with low “heterologous biases” and variability across samples (84.56 ± 0.98% and 85.63 ± 1.10, respectively).

Annotation completeness in terms of gene content of de novo transcriptome assembled with DRAP was assessed with BUSCO for the presence/absence of the conserved eukaryotic single copy orthologous genes. To refer to our previous work [21], completeness of transcriptome assembly using mapping approach with Cufflinks and BGI_duck_1.0 as reference genome was also assessed. As shown in Table 2, DRAP de novo approach produced more orthologues (97.1% completeness), provided a more complete (0% missing) and less fragmented (only 3%) catalog of orthologues when compared to the mapping approach using Cufflinks (81.5, 7.3 and 11.2%, respectively). Same BUSCO analyses were also conducted on BGI_duck_1.0 genome assembly and on two more recent genomes assembled at the chromosome level and expected to be more complete, i.e. CAU_duck1.0 (GCA_002743455.1) and ASM874695v1 (GCA_008746955.1) (Table 2). These three genomes contain more complete and single copy orthologous genes (81.2, 82.8 and 90.1%, respectively) than de novo transcriptome assembly with DRAP (59.1%) but less complete orthologues in total (82.9, 83.5 and 90.8% versus 97.1%) with more genes missing (7.2, 7.3 and 6.9% versus 0.0%) suggesting that our RNA sequencing and de novo transcriptome assembly allowed identifying new genes that are missing in genome assemblies.

**Table 2:** Assembly assessments with BUSCO

Categories	DRAP	Cufflinks	Reference genome		
			BGI_duck_1.0	CAU_duck1.0	ASM874695v1
Complete	97.1%	81.5%	82.9%	83.5%	90.8%
Complete and single-copy	59.1%	42.9%	81.2%	82.8%	90.1%
Complete and duplicated	38.0%	38.6%	1.7%	0,70%	0.7%
Fragmented	3.0%	11.2%	9.9%	9.2%	2.3%
Missing	0.0%	7.3%	7.2%	7.3%	6.9%

### Gene expression analyses

Gene expressions after DRAP de novo approach were analyzed with edgeR. Significant (*p*-value < 0.01) differentially expressed genes (fold change ≥2) were determined. In total, 13,898 different genes were found up- or down-regulated by overfeeding in the four genetic types (Table 3). Much less differentially expressed genes (DEG) were identified in Pekin than in other genetic types (3749, versus 6167, 8920 and 7696, respectively). Among all 13,898 DEG, 903 were identified in the four genetic types. Similar results were found when analyses were conducted with DESeq2 (data not shown).

**Table 3:** Differentially expressed genes using DRAP de novo approach

DEG	Pekin	Muscovy	Mule	Hinny	common
up	2281	3450	4907	3901	539
down	1468	2717	4013	3795	364
all	3749	6167	8920	7696	903

**Fig. 3:**
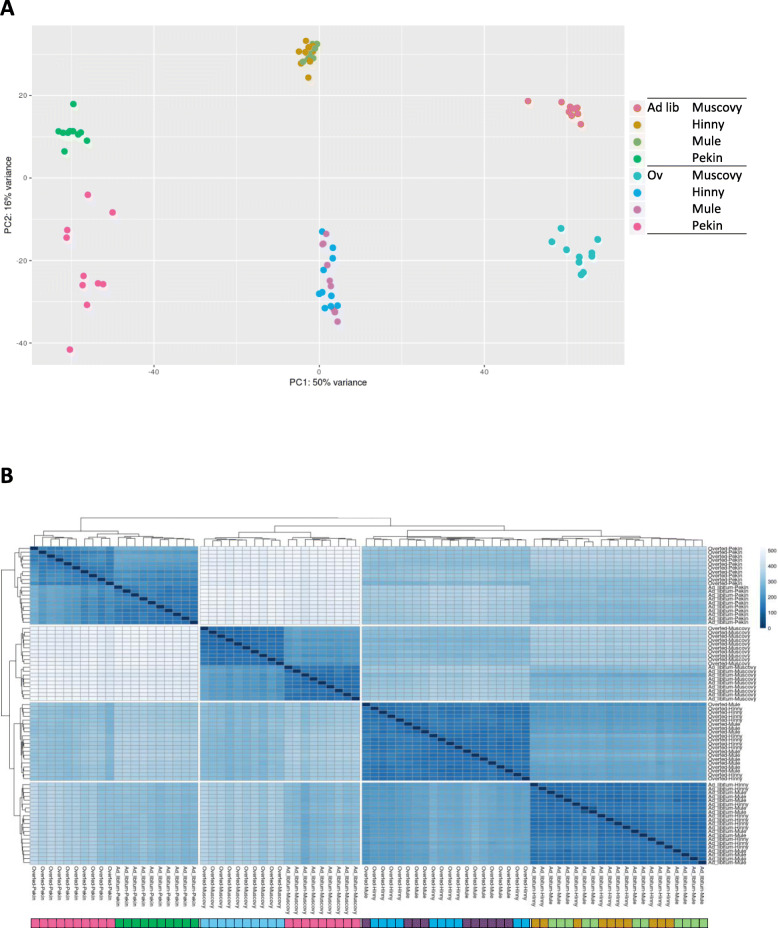
Clustering according to DEG expression levels. Expression levels of DEG were analyzed by **a** principal component analysis (PCA) and **b** hierarchical clustering (HC). Sample color code in (**b**) is the same as in (**a**)

Expression levels of DEG were analyzed by principal component analysis (PCA) and hierarchical clustering (HC) to cluster samples according to similarities in gene expression. As shown in Fig. 3a, the first principal component (PC 1) of PCA summarized 50% of the whole variability and discriminated samples according to genetic type, pure species being extreme and hybrids intermediate. The second principal component (PC2) summarized 16% of the whole variability and discriminated samples according to feeding. The cluster corresponding to overfed Pekin ducks appeared more dispersed than the other clusters. Two clusters were defined for Mule and Hinny samples according only to feeding (ad libitum and overfed) without any distinction according to genetic type. When expressions of DEG were analyzed by HC (Fig. 3b), two clusters were first defined: one including all Pekin samples, whatever the feeding was, and the other including all other samples. This second cluster included two groups. One corresponded to ad libitum and overfed Muscovy samples. The second included two groups: on one hand ad libitum and on the other hand overfed mule and hinny hybrid samples as was observed in PCA. Finally, 4 different clusters were defined again according first to genetic type: a Pekin cluster more distant from the 3 others and including ad libitum and overfed samples; a Muscovy cluster also including ad libitum and overfed samples; a fed ad libitum Hinny and Mule cluster; and an overfed Hinny and Mule cluster. As observed in PCA, these two latter clusters suggest that Hinnies and Mules in the same feeding status cannot be distinguished according to differential gene expression.

**Fig. 4:**
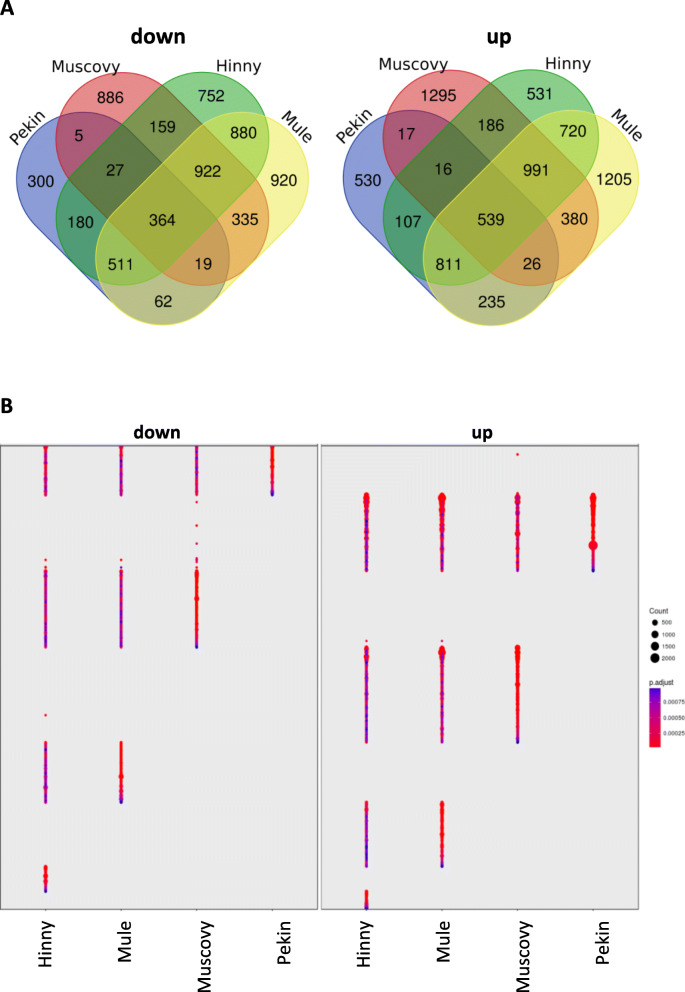
Differentially expressed genes. **a** Venn diagram of down and up-regulated DEG in the four genetic types. **b** Expression profiles of 903 DEG found in the four genetic types (fold changes FC > 2, adjusted *p* value < 0.05)

The presence of down- and up-regulated DEG in the four genetic types was shown with Venn diagrams (Fig. 4a). Few down- and up-regulated DEG (364 and 539, respectively) were identified in the four genetic types. However, fold change in expressions of these common DEG were very different according to genetic type (Fig. 4b). DEG expression levels in Pekin were more different from the three other genetic types with less down- and up-regulated DEG visualized, suggesting that DEG responses to overfeeding are less important in Pekin ducks. Conversely, many DEG were not found in each of the 4 genetic types or specifically found in one genetic type only (821, 2171, 1281, and 2125 in Pekin, Muscovy, Hinny and Mule ducks, respectively) indicating genetic type effects (Fig. 4a). Some examples taken at random of such feeding and genetic type effects interactions are shown in Additional file 1. They illustrate different types of response to overfeeding (fold change and/or up and down regulation) between the 4 genetic types. Hybrids shared the highest number of DEG with 2677 down- and 3061 up-regulated DEG found in both mule and hinny ducks. Only two genes were found with interaction, i.e. down-regulated in one hybrid and up-regulated in the other hybrid. These results indicate that few differences were observed in response to overfeeding between the two hybrids or in other words few genetic type effects and therefore few interactions with feeding effect. For comparison, less down- and up-regulated DEG were found in both mule and Muscovy ducks (1640 and 1936, respectively), even less in both mule and Pekin ducks (956 and 1611, respectively) and only 415 down- and 598 up- regulated DEG in both Pekin and Muscovy ducks indicating more differences in feeding effect between the two duck “pure” species.

### Functional enrichments

**Fig. 5:**
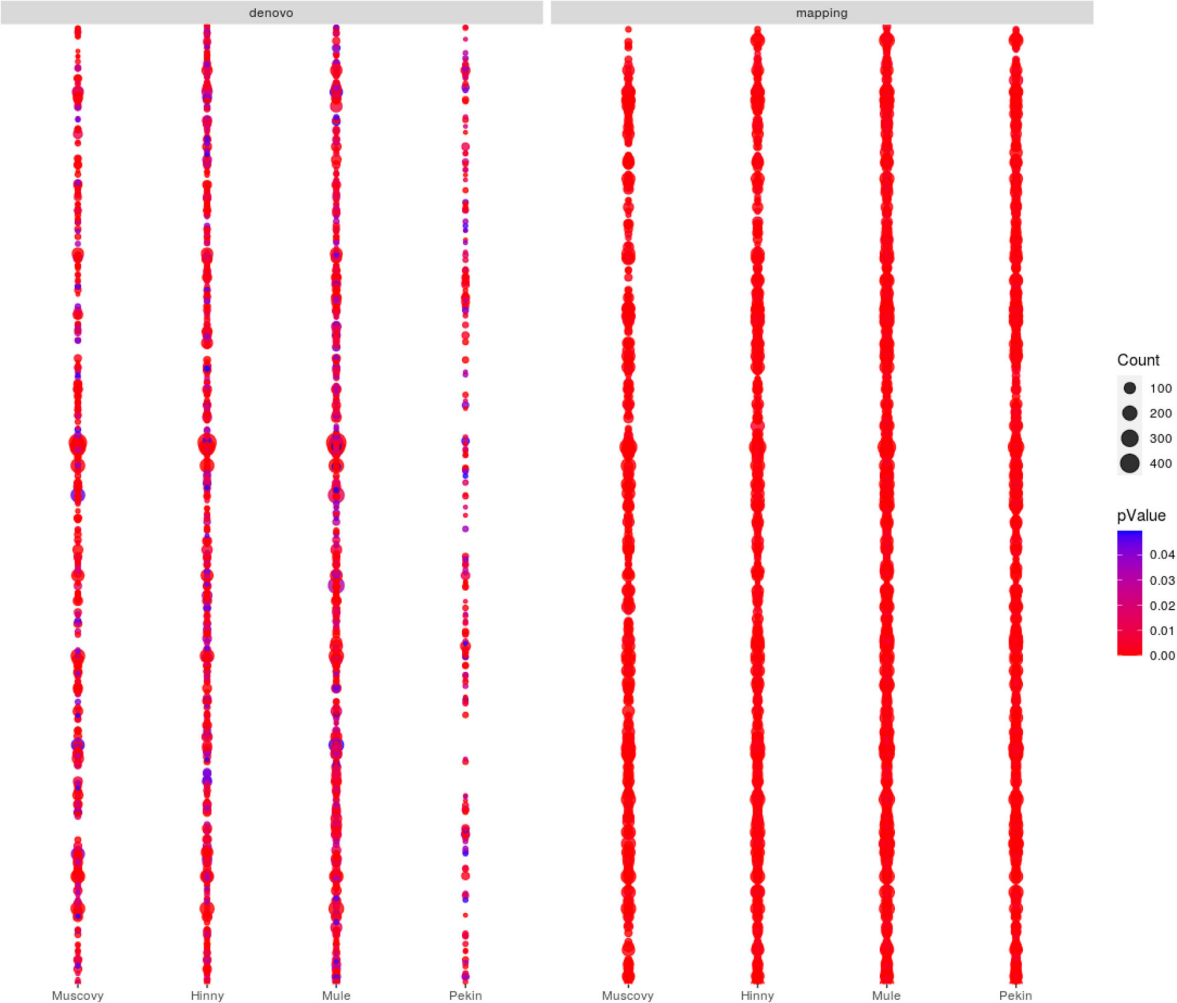
Enriched annotation profiles of DEG. Dot representation of significant (*p* < 0.05) enriched GOBP terms associated to differentially expressed genes (DEG) identified after de novo method (left panel) and reference based method (right panel). Dot size in profiles (counts) corresponds to number of DEG annotated with the GO term. *P*-values are shown in color bar, values decrease from more (red) to less significant (blue)

Functional enrichments in GOBP associated to DEG identified after DRAP de novo method were analyzed and compared to those identified after reference based method with BGI_duck_1.0 genome as conducted previously [21]. Many GOBP enrichments (535) were found whatever the method used (Additional files 2 and 3A). However, some differences were observed between methods as evidenced with enrichment annotation profiles (Fig. 5). Enrichments were less important with de novo method. Difference between Pekin and the 3 other genetic types was more important with de novo method and many GOBP enrichments found with reference based method (744) were not found with DRAP method (Additional file 3C). Interestingly, many GOBP were found enriched with DRAP method (1257), but most of them corresponded to few DEG (Additional file 3B) and/or were found in 1 or 2 genotypes only (Additional file 2).

**Fig. 6:**
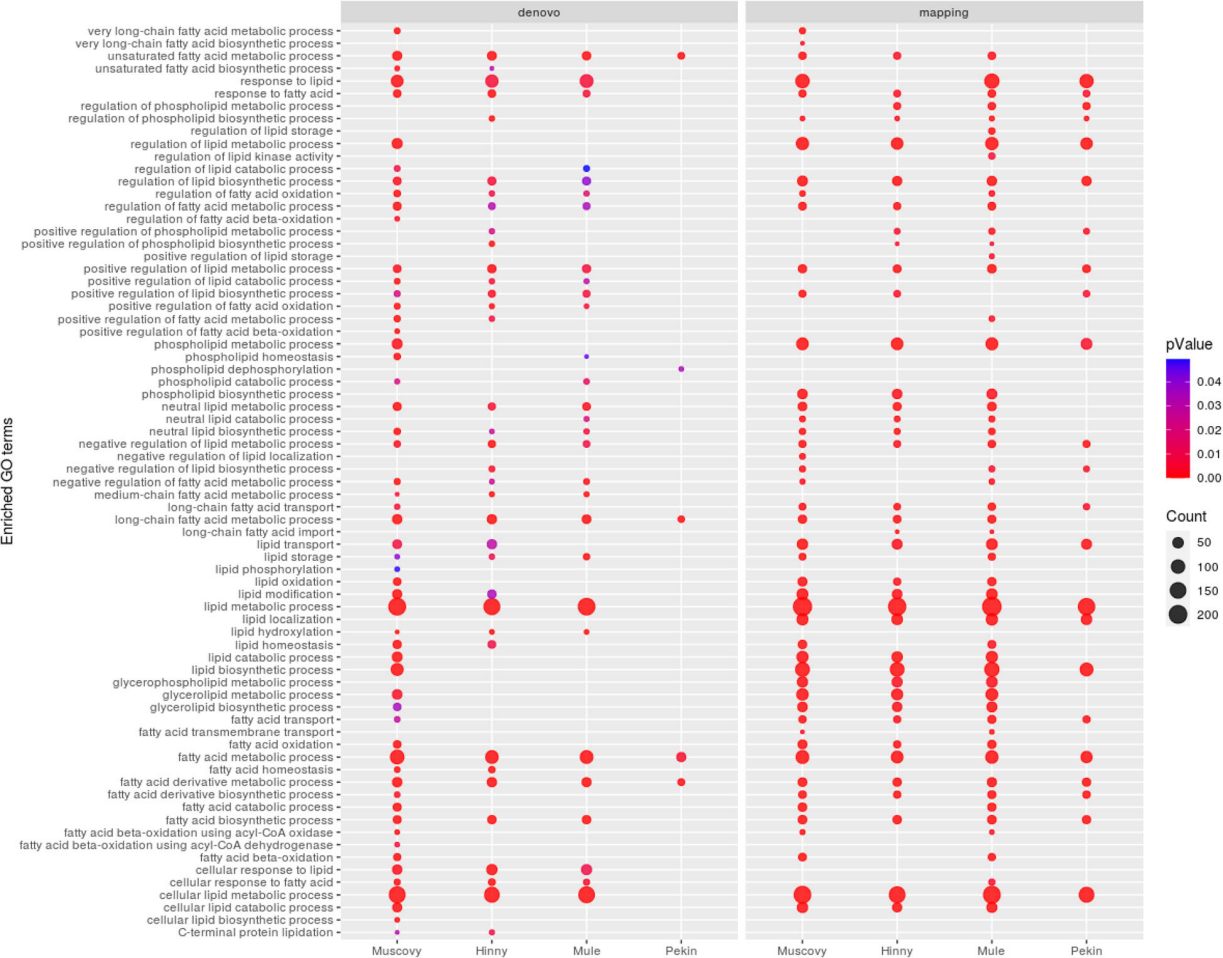
Lipid and fatty acid enriched annotation profiles. Dot representation of significant (*p* < 0.05) enriched GOBP terms related to lipid and fatty acid and associated to differentially expressed genes (DEG) identified after de novo method (left panel) and reference based method (right panel). Dot size in profiles (counts) corresponds to number of DEG annotated with the GO term. *P*-values are shown in color bar, values decrease from more (red) to less significant (blue)

As expected, most of the enriched biological processes found with the 2 methods were related to metabolism of lipids but also to many other processes related to mitosis and cell cycle, transmembrane transport, ion homeostasis and inflammatory response to cite the more enriched (Additional file 2). To better characterize lipid metabolism, we focused on GOBP terms including lipid or fatty acid words. As shown in Fig. 6, metabolism and regulation of fatty acids and neutral lipids, lipid signaling and response to lipid biological processes were enriched in Muscovy ducks and the two hybrids and much less or not in Pekin ducks. Again, difference between Pekin and the 3 other genetic types was more important with de novo method.

## Discussion

The aim of this study was to analyze and compare gene expressions in four different duck genetic types, common Pekin and Muscovy duck species and their reciprocal mule and hinny hybrids using a relevant approach. As mentioned by Moreton et al. [14], choice for using reference-based or de novo transcriptome assembly approach for gene expression analyses is generally based on the question whether or not a reference genome is available. However, when gene expressions from different species are to be analyzed and compared, the question of strategy is more complex and cannot be taken up with the question of availability of a reference-genome and neither strategy seems a priori better than the other.

In a previous study, we have conducted a reference-based approach using common duck (*Anas platyrhynchos*) BGI_duck_1.0 genome assembly as reference for the four genetic types [21]. As indicated, mapping rates were very different when homologous and heterologous mapping were considered (71% with Pekin duck reads and 41% with Muscovy duck reads). In the study of Wang et al. [18], homologous mapping rate of horse reads on horse reference genome (61.70%) was very similar to heterologous mapping rate of donkey reads on horse reference genome (62.07%). The reason of this difference of heterologous mapping rate between equids and ducks was not directly investigated but we can speculate that it is partly due to the difference of evolution distance between equids and duck species (estimated time according to http://www.timetree.org/, [29], 7.7 and 22.8 MYA, respectively), but also to different mapping parameters in the two studies. In our study, these parameters were set by defaults and were not optimized for heterologous mapping. As described in our previous study [21], this bias of mapping was not a problem to analyze differences in gene response to feeding in ducks from the same genetic type. It was however too important for gene expression comparisons between the four genetic types and to analyze genetic effect in gene expression.

Therefore, de novo transcriptome assembly approaches were then conducted. As shown in Table 1 and Fig. 1a, Trinity “single” transcriptome assemblies from Pekin or Muscovy duck sequences were very similar and better in terms of length and alignments of reads than assemblies from hinny and mule hybrid sequences. This result is probably due to the fact that in hybrids transcripts are expressed from two different genomes making assembly less efficient according to the presence of some polymorphisms in transcripts from the same orthologs between the two species. This was also observed when assemblies were performed with sequences from two different species (Fig. 1b). We have also shown that more reads were aligned on these de novo assembled transcriptomes than on common duck reference genome BGI_duck_1.0. Furthermore, homologous mapping of Pekin reads on de novo Pekin transcriptome assembly (around 85%) was more efficient than that on reference genome (71%). We can speculate that some reads correspond to genes that are missing in this reference genome, corresponding to new or misassembled genes, or to genes that are in unknown regions, i.e. unassembled and unassigned regions. Thus, these reads are not mapped on reference genome but are assembled together in transcripts by de novo approach and map on theses transcripts.

As Trinity and Oases were not well suited for “complex” transcriptome assembly, we have tested de novo RNA-Seq Assembly Pipeline (DRAP) [28] to perform a meta-transcriptome assembly of transcriptomes from the four duck genetic types. As shown in Fig. 2, this meta-transcriptome assembly with DRAP was very efficient, with a mapping rate of sample reads similar to the best rates previously observed, i.e. homologous mapping rate of Pekin or Muscovy duck reads on de novo assembled Pekin or Muscovy transcriptomes. Further analysis with BUSCO [30] confirmed the quality of this meta-transcriptome assembled with DRAP (Table 2). In our study, more than 97% of expected single-copy orthologues were assembled by DRAP as complete (i.e. full expected transcript length), 3% were fragmented (assembled with a shorter length than expected) and finally no (0%) were missing. With reference-based approach using Cufflinks and BGI_duck_1.0 assembly, only 81.5% single-copy orthologues were assembled to full length, 11.2% were fragmented and 7.3% were missing. This clearly indicates that de novo assembly of meta-transcriptome using DRAP is of higher quality when compared to assembly using reference-based approach as performed earlier [21]. Again, this is probably due to the missing of genes in thisgenome. Moreover, when BUSCO was applied directly to this genome, proportions of complete and missing orthologues were similar (82.9 and 7.2%, respectively) to those found after reference-based approach for transcriptome assembly using Cufflinks. BUSCO analyses were also conducted on CAU_duck1.0 and ASM874695v1 common duck genomes assembled more recently. As expected, completeness of these two genomes was more important (83.5 and 90.8%, respectively). However, completeness were lower than that of DRAP transcriptome assembly and no improvement on missing orthologues was observed (7.3 and 6.9%), again suggesting that de novo transcriptome assembly with DRAP was more complete. It is interesting to note that lower proportions of complete and single copy orthologs were found in DRAP assembled transcriptome and Cufflinks transcriptome assembled with BGI_duck_1.0 genome (59.1 and 42.9%) than in all three different assemblies of the genome (81.2, 82.8 and 90.1%). The fact that most of orthologues were complete and as expected in a single copy in the genome and not in transcriptomes whatever the approach used is probably the result of the presence of only one isoform for one orthologue in the genome (by definition because BUSCO applies only on single-copy orthologues in related species) but of multiple alternative transcripts from one single-copy orthologue making assembly in one transcript more tricky. This clearly indicates that development of methods to improve transcriptome assembly and reduce duplication and more generally redundancy remains a concern today. Nevertheless, our results show that the de novo approach with DRAP is more relevant than the reference-based approach to assemble a meta-transcriptome from reads of the four duck genetic types.

Gene expression analyses were then conducted using this meta-transcriptome as reference, assuming that mapping bias were substantially reduced and allowed us to analyze DEG within and between genetic types. Many genes were found up-and down-regulated by overfeeding and some of them (903) were found in the four genetic types. These results indicate some similarities but also some genetic type-specific responses to overfeeding. This was further confirmed by principal component analyses and hierarchical clustering, clearly showing that Pekin ducks are different from the three other genetic types.

Functional enrichments in GO biological processes were finally conducted on DEG identified by DRAP de novo method and compared to those of DEG found with reference based method using BGI_duck_1.0 genome. Most of the most enriched biological processes identified after reference based method, notably lipid metabolism, were also observed with DRAP method, indicating that most of our previous conclusions remain valid when using de novo method. However, it is important to note that enrichments appeared overestimated with reference based method when compared to de novo method. According to mapping rates on BGI_duck_1.0 genome and DRAP meta transcriptome, we assume that de novo method is more relevant to compare DEG and associated functions between the four genetic types. It would have be interesting to compare the two methods using a more complete duck genome assembly, ASM874695v1 for example, to apply the reference based method. However, as this genome is less complete than meta-transcriptome assembled de novo and more importantly is always fully specific to Pekin duck only, we think that mapping biases would be similar to those observed with BGI_duck_1.0 genome and the results as well.

Many enriched biological processes specifically found with DRAP method were represented by very few DEG (Additional file 3B). It is difficult to draw definitive conclusion about this result without further studies on related genes. Conversely, no unexpected biological process was highly enriched and specifically found using DRAP method.

Different conclusions can be drawn from these results. First, responses to overfeeding in hinny and mule hybrids were very similar and indicate that expectation that hinnies are not used for fatty liver production due to a different and lower response to overfeeding is wrong. In fact, the reason essentially lies in the difficulty to produce viable hinnies due to a lower efficiency of artificial insemination of Muscovy females and their lower fertility when compared to Pekin females. Second important conclusion is that many genes are differentially expressed in the four genetic types, including those related to lipid metabolism, but also related to many other biological processes. As previously observed [21], this suggests that response to overfeeding and consequently development of hepatic steatosis is a complex trait involving many genes and functions and is not well described by the expression of few candidate genes [6–9], even if expressions of these genes are well correlated to fatty liver weight [6]. Third, although some differentially expressed genes were found in the four genetic types, some other were not found in Pekin ducks or expressed at a lower level, including those involved in fatty acid and lipid metabolism making this species less similar and more distant to the three other genetic types. Furthermore, individual variability of responses in Pekin ducks was very important making some overfed Pekin samples near to Pekin ducks fed ad libitum samples. This is directly linked to the greater variability and lower ability of Pekin ducks to produce fatty liver in response to overfeeding when compared to hinny and mule hybrids and Muscovy ducks. To conclude on this point, response to overfeeding in ducks is very complex, different in some extent from a genetic type to the other and involves many genes suggesting that genetic improvement of duck selection for fatty liver production should be undertaken at the whole genome level.

## Conclusions

We have shown that differential gene expression analyses in different duck genetic types and comparisons are more accurate when a meta-transcriptome is assembled with DRAP from single transcriptomes of the four different genetic types assembled by Trinity. Using this strategy, we have shown that differences in responses to overfeeding can be identified between the four genetic types and that Pekin ducks greatly differ from ducks of the 3 other genetic types. This work provides new information to describe hepatic steatosis in different duck genetic types in a much more holistic way. This information could also be exploited in the context of genomic selection programs that will certainly be developed in ducks in the coming years.

## Methods

### Animals and experimental design

Animals, experimental design, RNA preparation and sequencing have been described in previous publications [5, 6, 8, 21]. Briefly, male ducks corresponding to common Pekin (*Anas platyrhynchos*) and Muscovy (*Cairina moschata*) duck species and to their two reciprocal interspecific Mule and Hinny hybrids were fed ad libitum or overfed 14 days with corn and corn meal at Experimental Station for Waterfowl Breeding (INRA, Artiguères, France). After electronarcosis, ducks were euthanized by neck sectioning and liver samples were collected and frozen in liquid nitrogen [5]. RNA were extracted from liver samples [6], cloned in libraries and paired-ends sequenced [21].

The whole transcriptomic project included 80 Illumina libraries for a total of 2,252,041,541 paired-end reads with a read length of 100 bp. The four genetic types (*Anas platyrhynchos*, *Cairina moschata*, Hinny and Mule hybrids) correspond to 20, 19, 20 and 21 libraries for 554,893,036, 513,608,258, 544,599,927 and 638,940,320 paired end reads in total for each of them, respectively. RNA sequences are accessible in the NCBI sequence read archive (SRA) under the accession number SRP144764.

### Transcriptome assembly using reference genome

After adapter trimming, mapping of RNA sequences and transcripts reconstruction on the common duck (*Anas platyrhynchos*) reference genome BGI_duck_1.0 (INSDC Assembly GCA_000355885.1) [27] were performed using TopHat2, Cufflinks and Cuffmerge tools of the Tuxedo suite [17] as previously described [21].

### De novo transcriptome assemblies

RNA-seq data were alternatively assembled using de novo approaches. The raw datasets for each of the 4 species were assembled independently using Trinity v2.3.2 [22, 31], including a reads cleaning process with Trimmomatic (using defaults parameters) [32] and an in silico reads normalization step (provided within the Trinity suite). Parental transcriptome (mixing Pekin and Muscovy normalized reads) and hybrid transcriptome (mixing Hinny and Mule duck normalized reads) were assembled with Trinity and Oases (−m 25 -M 65 -s 10) [23]. Composite meta-transcriptome assembly of single genetic type transcriptomes was also conducted using DRAP v1.91 [28] for compacting and correcting Trinity assemblies and constructing a reference transcriptome. By default, DRAP produced 4 assemblies which were filtered for low coverage with Fragments Per Kilobase per Million (FPKM) thresholds set at 1, 3, 5 and 10 respectively.

### Assembly assessment

Quantification for the abundances of transcripts was performed by library using pseudo-aligner Kallisto [33] (trinityrnaseq-2.4.0/util/align_and_estimate_abundance.pl) in no strand-specific paired-end mode. Transrate [34] assembly evaluation which was regrouped in DRAP package was set to version 1.0.1. Transcriptome completeness was assessed by BUSCO v3.0.2 [30] with Eukaryota sets odb9.

### Functional annotation of transcripts

Peptide prediction was performed using TransDecoder 5.0.2 [31]. Similarity search (Blast search using Atomic Blast+, https://github.com/ppericard/bioinfo-toolkit/blob/master/bin/atomicblastplus.py v5.0.2) of transcriptome and the Transdecoder predicted peptides were performed against the uniprot-swissprot databases (provided by Trinotate annotation pipeline v3.1.1: june 2017). Peptide signal prediction was performed using signalP v4.1 (−c 0) [35]. Prediction of transmembrane helices in proteins was performed using TMHMM v2.0c [36]. Protein domain search was performed using hmmscan from the HMMER v.3.1b2 suite against the Pfam-A database (provided by Trinotate) [37]. Identification of rRNA sequences was performed using RNAmmer v1.2 [38]. Finally, transcriptome functional annotation was performed using Trinotate pipeline v3.1.1 (http://trinotate.github.io described in [39]).

### Differential gene expression and enrichment analyses

Differential gene expression analyses were performed using TMM [40] normalization counts at gene level with GO-enrichment at the same time by DEseq2 [41] and edgeR [42] through Trinity suite v2.5.1 (−-dispersion 0.1 -P 1e-3 -C 1). Common and specific DEG were described using Venn diagrams (http://bioinformatics.psb.ugent.be/webtools/Venn/). Expression profiles of down- and up-regulated DEG were drawn using clusterProfiler v3.5 [43]. GO biological process (GOBP) enrichments were analyzed with Webgestalt [44, 45].

## Supplementary information

**Additional file 1.** Examples of interactions between feeding and genetic type effect.

**Additional file 2.** GO Biological process enrichments in DEG data.

**Additional file 3.** Numbers of enriched GO terms as a function of DEG numbers found with reference based and/or DRAP methods.

## Data Availability

The dataset supporting the conclusions of this article is available in the NCBI Sequence Read Archive (SRA), under the accession number SRP144764 (https://www.ncbi.nlm.nih.gov/sra/SRP144764).

## Abbreviations

BUSCO: Benchmarking Universal Single-Copy Orthologs
BWA: Burrows-Wheeler transform
DEG: Differentially expressed gene
DRAP: De novo RNA-Seq Assembly Pipeline
FPKM: Fragments per kilobase per million
HC: Hierarchical clustering
NCBI: National Center for Biotechnology Information
PC: Principal component
PCA: Principal component analysis
RNA: Ribonucleic acid
RNA-seq: Ribonucleic acid sequencing
SRA: Sequence read archive
