# Supplementary material for: Development of a relevant strategy using de novo transcriptome assembly method for transcriptome comparisons between Muscovy and common duck species and their reciprocal inter-specific mule and hinny hybrids fed ad libitum and overfed
Source: BMC Genomics. 2020 Oct 2;21:687. doi: 10.1186/s12864-020-07099-4 (PMC7531116; doi:10.1186/s12864-020-07099-4)
Supplement: Supplementary file 1 — Additional file 1. Examples of interactions between feeding and genetic type effect. [file 12864_2020_7099_MOESM1_ESM.pdf]

Examples of feeding and gene effects interactions
FC: fold-change
p: adjusted p value
Red box: significant up-regulated gene
Green box: significant down-regulated gene
Grey box: insignificant (p > 0.05)

AVD (avidin)

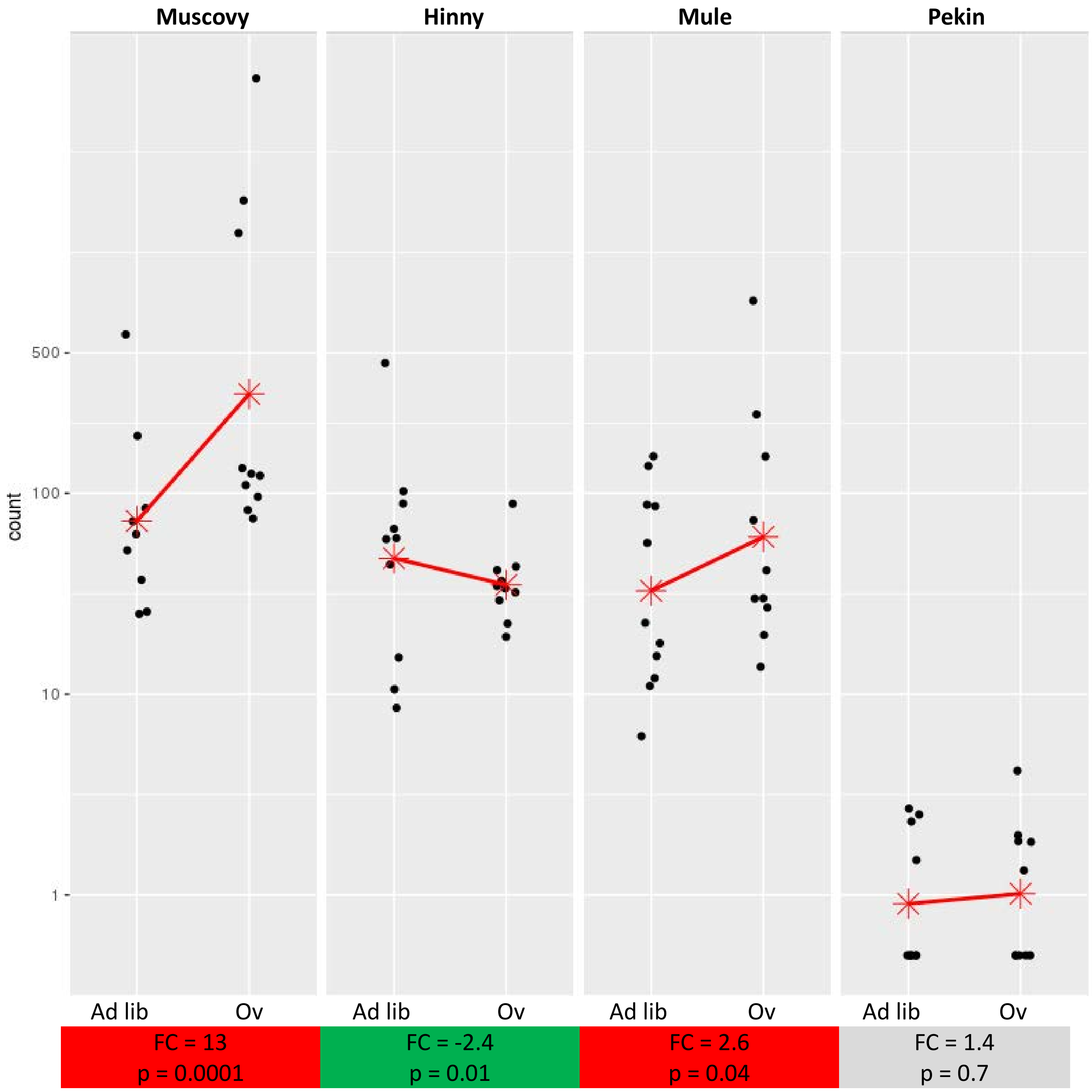

CYP4V2 (cytochrome P450 family 4 subfamily V member 2)

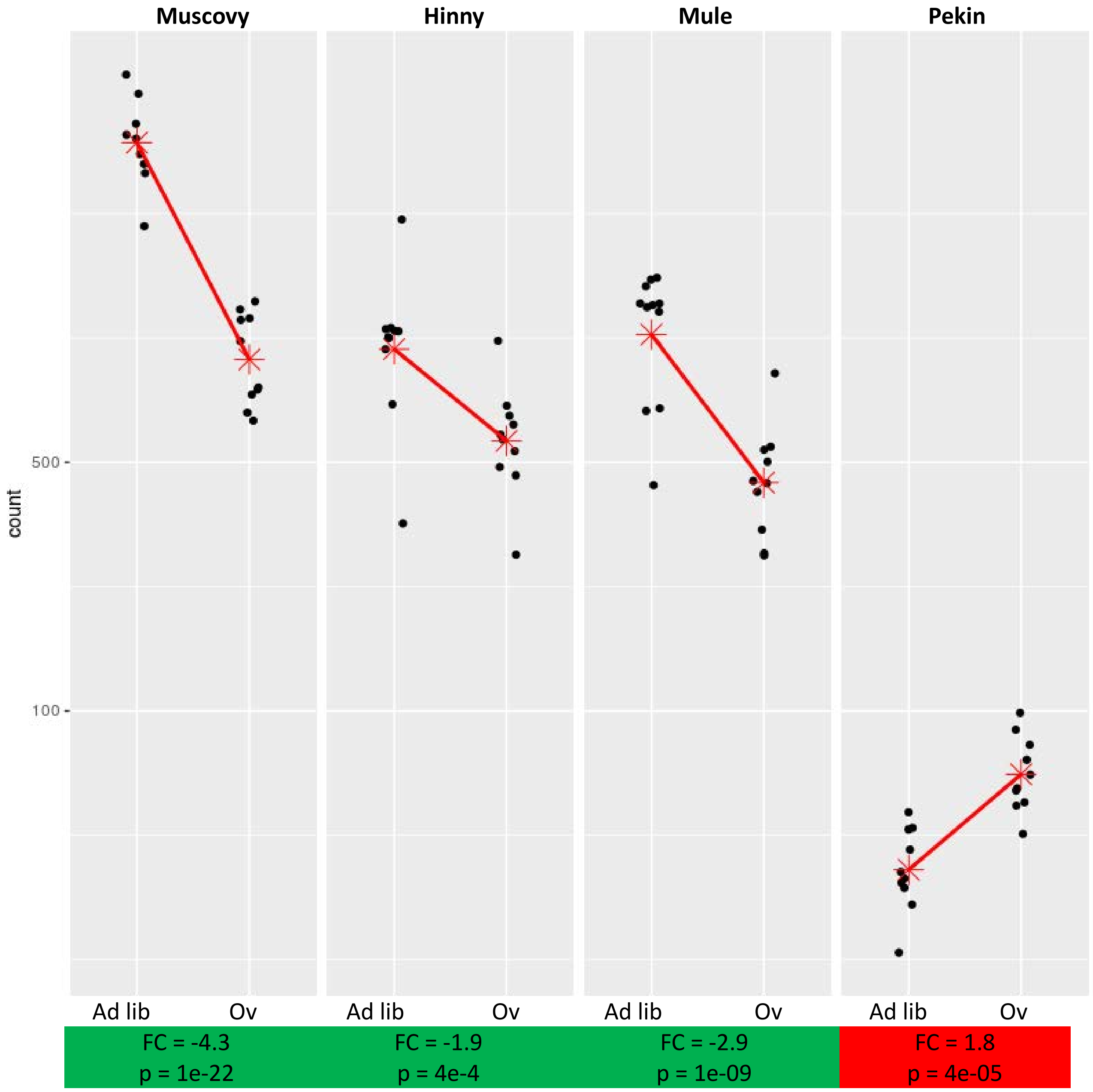

Muscovy	Hinny	Mule	Pekin
FC = -4.3<br>p = 1e-22	FC = -1.9<br>p = 4e-4	FC = -2.9<br>p = 1e-09	FC = 1.8<br>p = 4e-05

FTH1 (ferritin heavy chain 1)

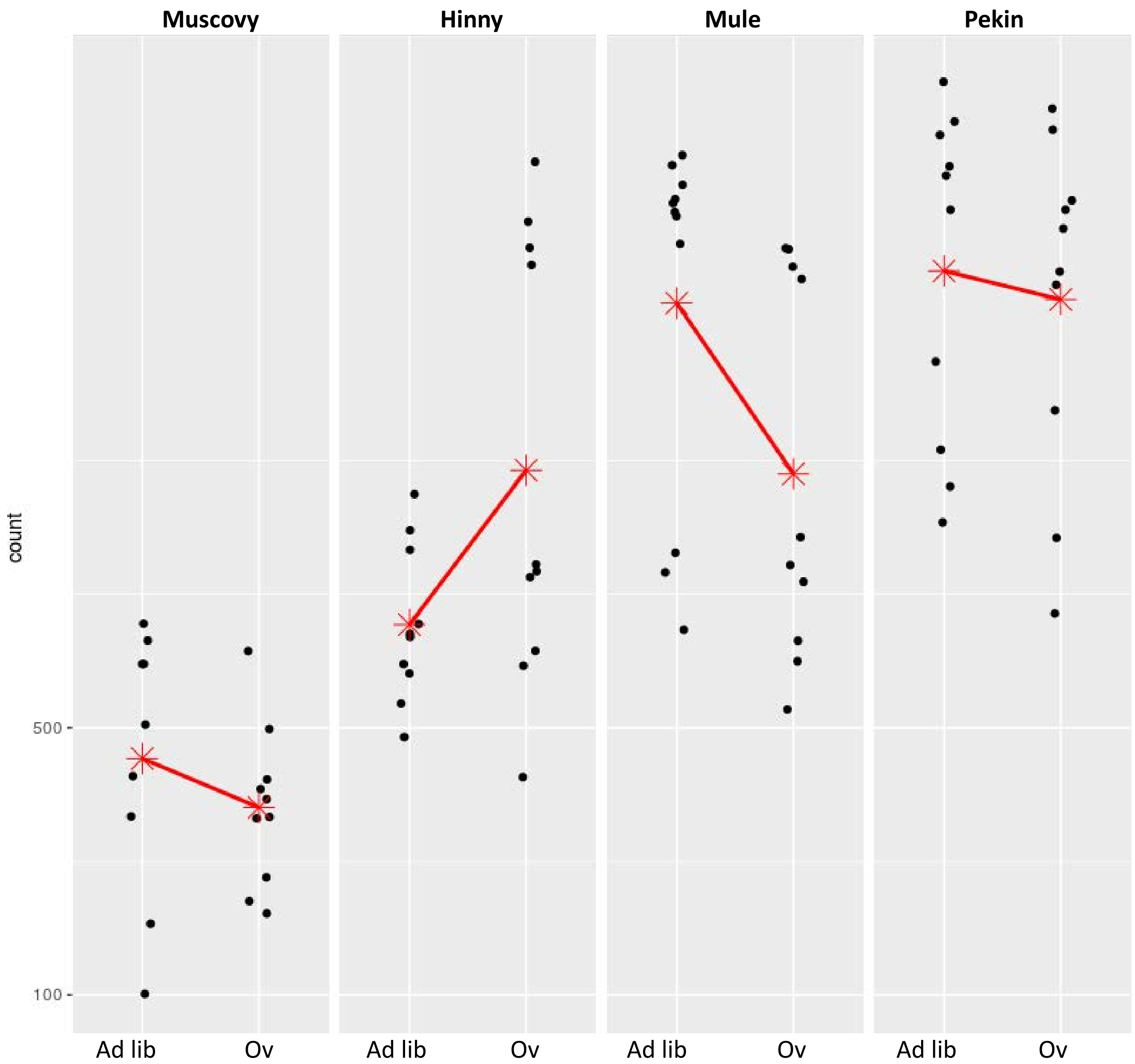

Muscovy	Hinny	Mule	Pekin
FC = -1.6<br>p = 0.09	FC = 4.5<br>p = 8e-05	FC = -2.5<br>p = 0.01	FC = -1.2<br>p = 0.7

LPL (lipoprotein lipase)

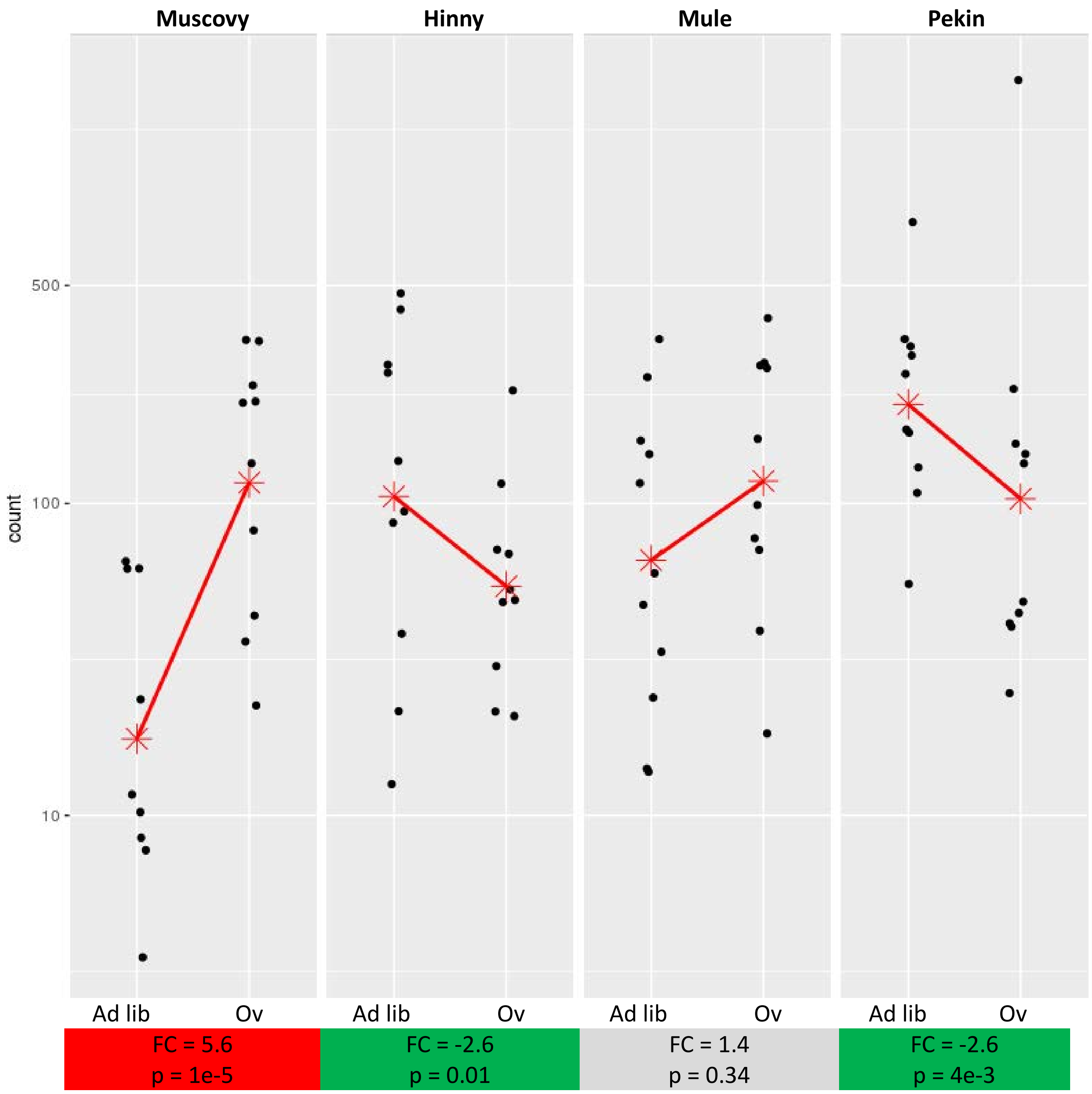

PCK1 (phosphoenolpyruvate carboxykinase 1)

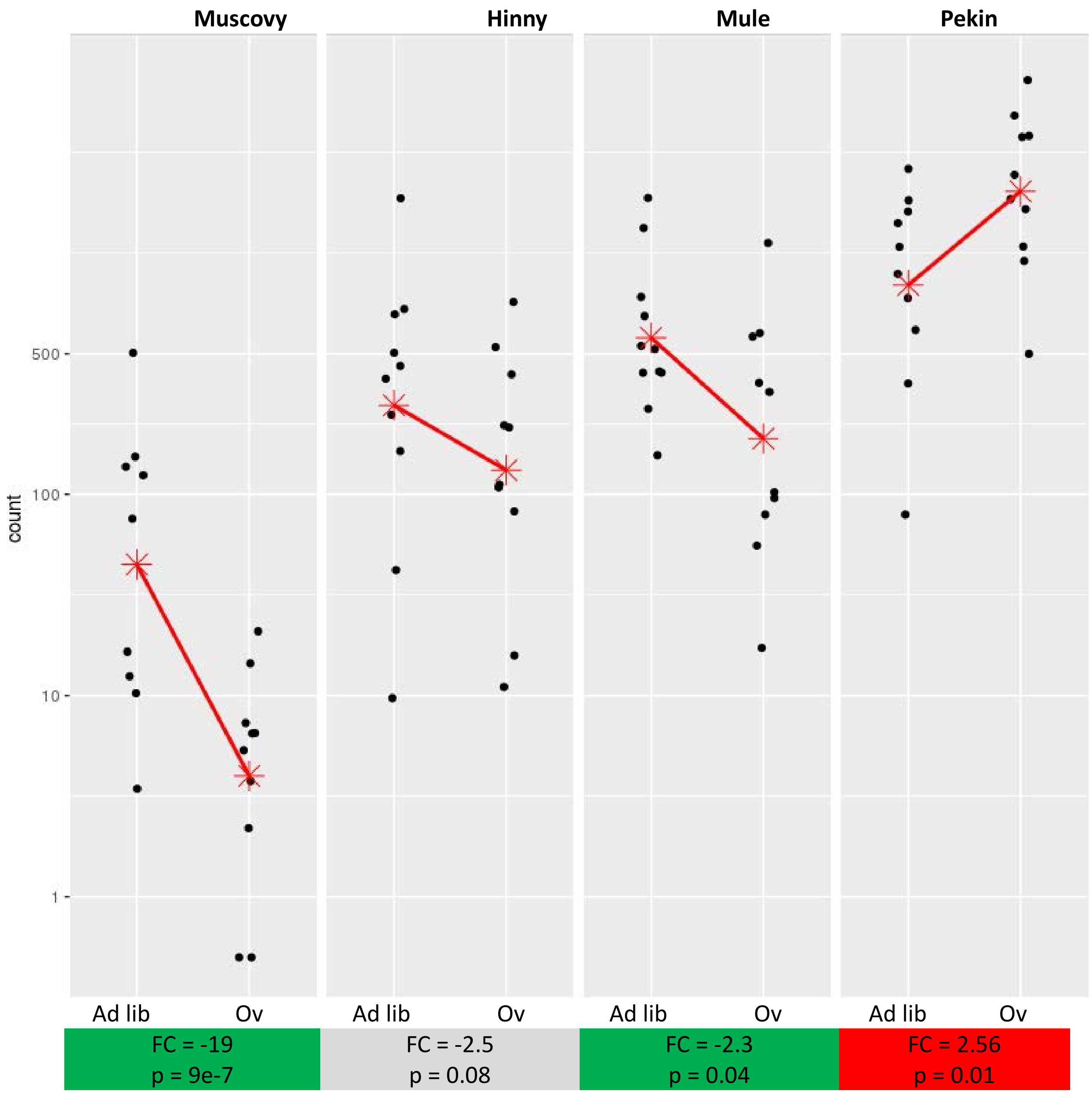

PFKFB3 6-phosphofructo-2-kinase/fructose-2,6-biphosphatase 3)

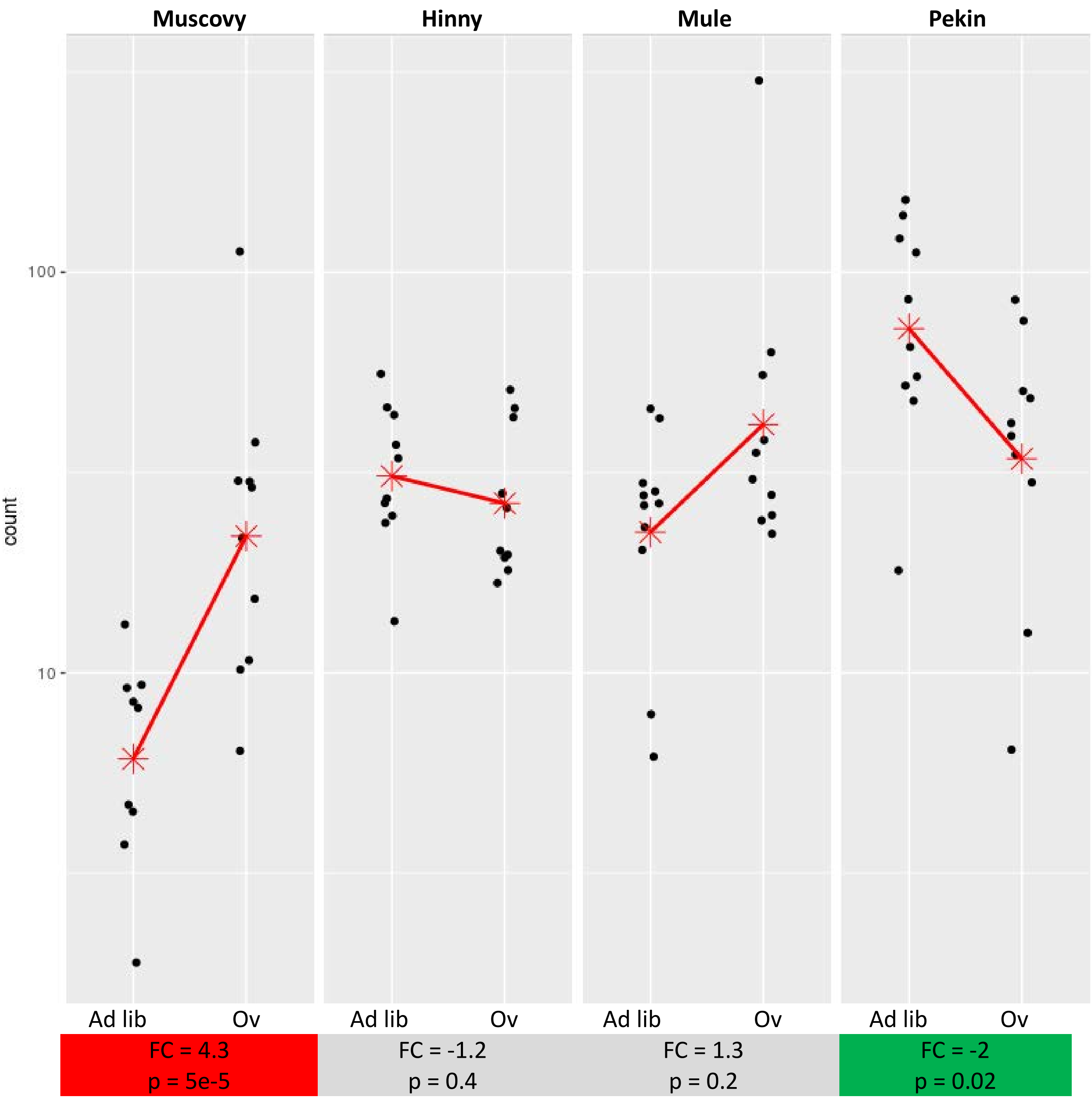

PLIN1 (perilipin 1)

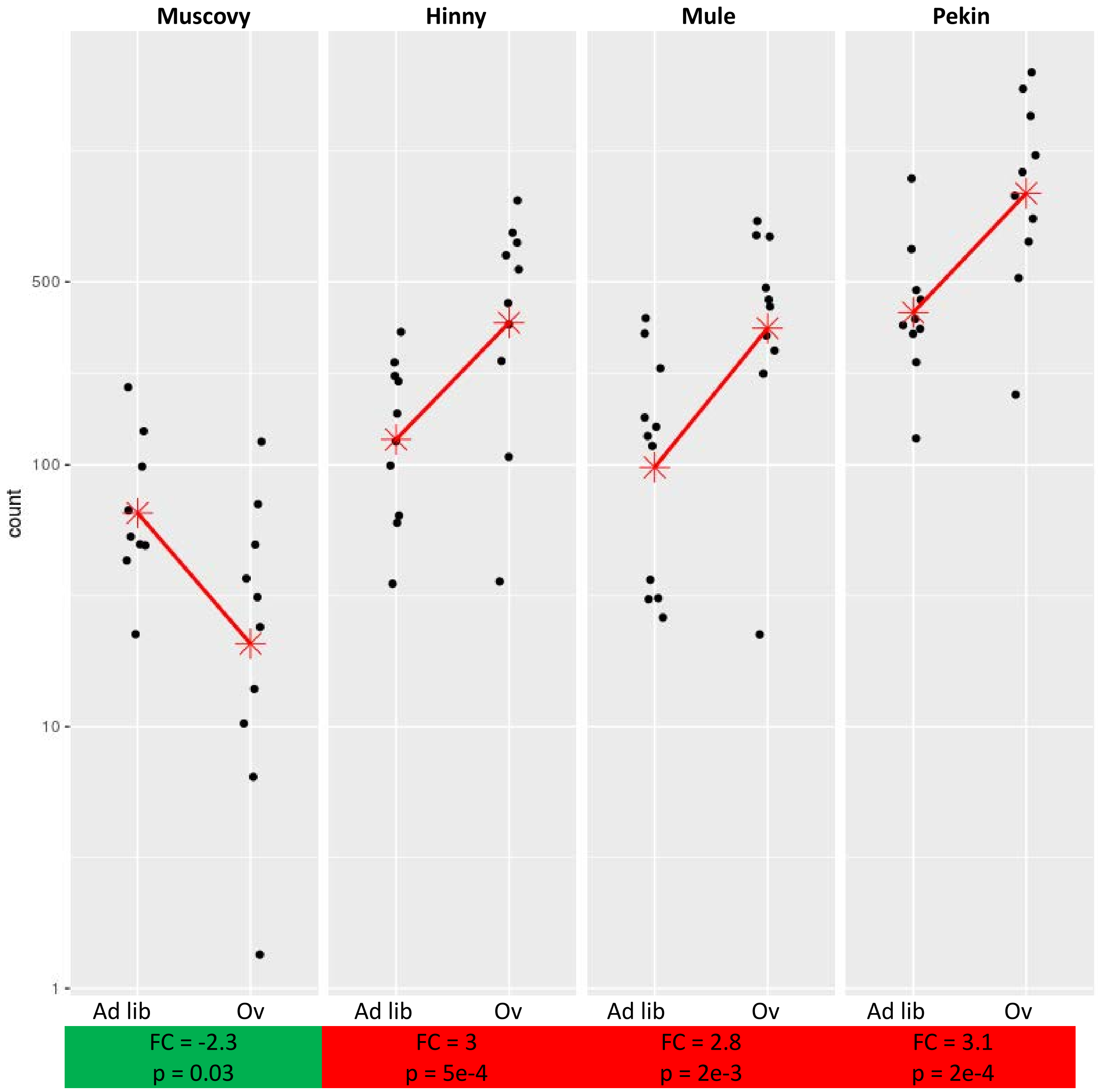

Muscovy	Hinny	Mule	Pekin
FC = -2.3<br>p = 0.03	FC = 3<br>p = 5e-4	FC = 2.8<br>p = 2e-3	FC = 3.1<br>p = 2e-4
