# Supplementary material for: Development of a relevant strategy using de novo transcriptome assembly method for transcriptome comparisons between Muscovy and common duck species and their reciprocal inter-specific mule and hinny hybrids fed ad libitum and overfed
Source: BMC Genomics. 2020 Oct 2;21:687. doi: 10.1186/s12864-020-07099-4 (PMC7531116; doi:10.1186/s12864-020-07099-4)
Supplement: Supplementary file 3 — Additional file 3. Numbers of enriched GO terms as a function of DEG numbers found with reference based and/or DRAP methods. [file 12864_2020_7099_MOESM3_ESM.pdf]

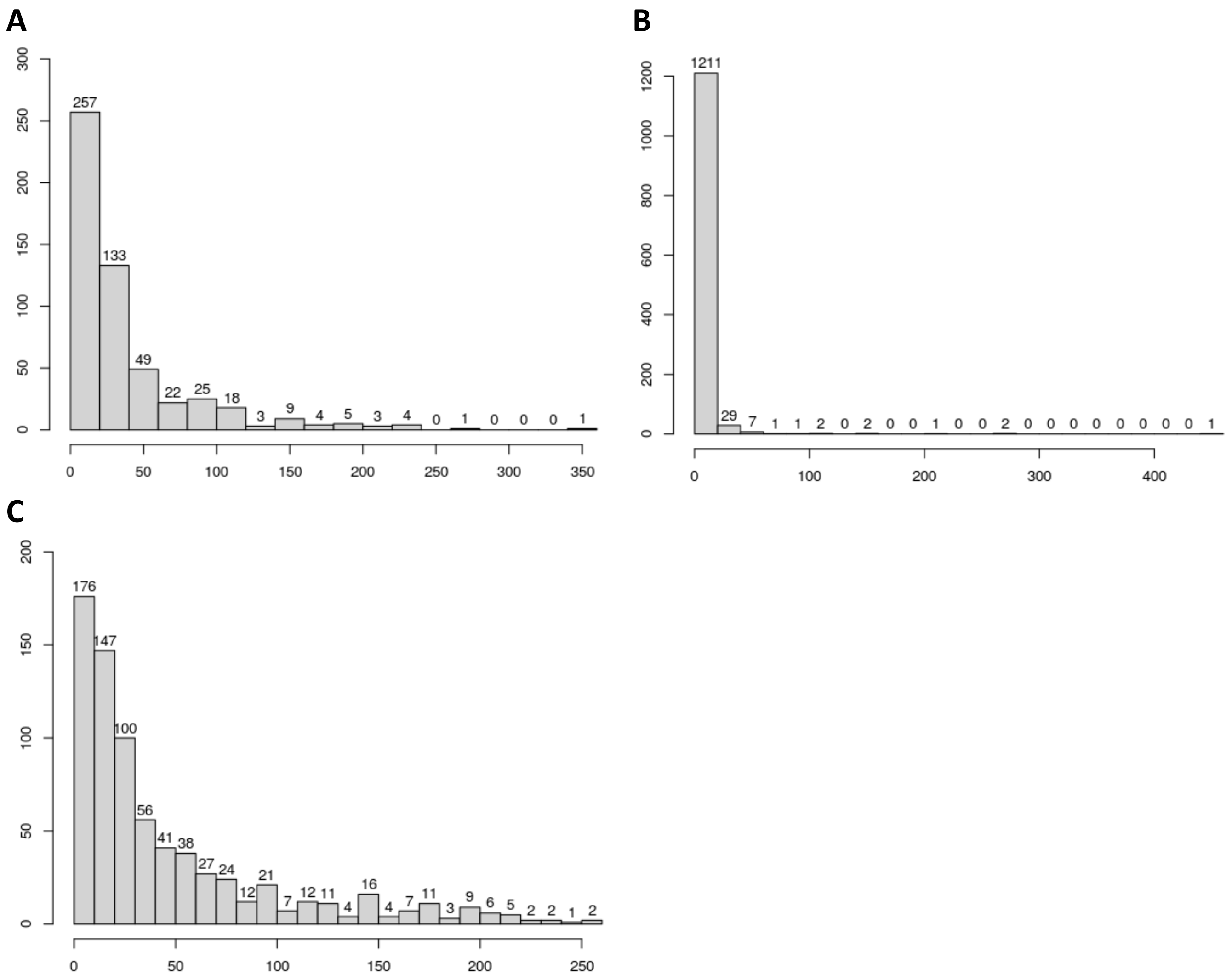

Numbers of enriched GO terms (y-axis) as a function of DEG numbers (x-axis) found with reference based and DRAP de novo methods (A), with DRAP de novo method only (B) or reference based method only (C).
